# The efficient measurement of individual differences in meaning motivation: The need for sense-making short form

**DOI:** 10.3389/fpsyg.2022.945692

**Published:** 2022-08-18

**Authors:** Katarzyna Cantarero, Wijnand A. P. van Tilburg, Agata Gasiorowska, Eric R. Igou

**Affiliations:** ^1^Social Behavior Research Center, SWPS University of Social Sciences and Humanities, Wrocław, Poland; ^2^Department of Psychology, University of Essex, Colchester, United Kingdom; ^3^Center for Research in Economic Behavior, SWPS University of Social Sciences and Humanities, Wrocław, Poland; ^4^Department of Psychology, University of Limerick, Limerick, Ireland

**Keywords:** need for sense-making, meaning, human motivation, basic psychological needs, individual differences, scale development

## Abstract

People differ in the extent to which they express a need for sense-making (NSM), and these individual differences are important to understand in light of meaning-making processes. To quantify this important variable, we originally proposed a need for sense-making scale. We now propose a refined, similarly reliable short version of the scale (NSM-SF). The 7-item NSM-SF was validated across a series of four studies (combined *N* = 1,243). NSM-SF showed psychometric properties and correlations consistent with its longer forerunner. Additionally, results indicated that the need for sense-making was moderately positively related to the satisfaction of basic psychological needs (autonomy, relatedness and competence), and it related negatively to the frustration of these needs. The research offers a useful, brief tool for assessing the NSM construct and broadens our understanding of basic psychological motivations.

## Introduction

Meaning-making processes and sense-making^1^ motivation have been in the spotlight of research on the psychological functioning of individuals for some time (e.g., Graeupner and Coman, 2017; Petrou et al., 2017; Iwasaki et al., 2018; Walsh, 2020). This scientific interest may be attributed to the finding that sense-making motivation is related to important outcomes across life domains (personal, organizational, and societal). For example, research shows that finding meaning in difficult events is beneficial to individuals as it relates to lower stress (Updegraff et al., 2008). Meaning-making motivation is also linked to how individuals experience their professional activities. For instance, the perception of meaningful work is related to work engagement (e.g., May et al., 2004). The latter, in turn, is linked to better work performance and organizational commitment (Schaufeli and Bakker, 2014). Additionally, meaning-making processes are potentially important for conservation behavior and sustainability. Perceptions of meaningfulness relate to conservation intentions through experiencing disappointment (Byrka et al., 2021). Research on sense-making thus suggests that it is an important part of human functioning, which has not only psychological consequences but may exert a broader societal impact.

## Individual differences in need for sense-making

The motivation to find meaning has been long argued to be central to human functioning (e.g., Frankl, 2006). Accordingly, a growing body of research has explored individual differences related to meaning-making processes. Baumeister (1991) proposed differentiating between lower, more concrete meaning versus higher, more abstract meaning. The latter of which has often been investigated in the context of meaning in life (e.g., Steger et al., 2011; Abeyta and Routledge, 2018). Steger et al. (2006) developed the Meaning in Life Questionnaire to assess both the presence and search for meaning in life. They found that perceiving one’s life as meaningful is more typical among those with high self-esteem levels and who are satisfied with life. Researchers also showed that searching for meaning in life tends to be negatively related to presence of meaning in life (e.g., Steger et al., 2006). There have also been studies pointing to individual differences in the need to have a meaningful life, which is related to, for example, religious commitment and beliefs (Abeyta and Routledge, 2018). Both the MLQ and need for meaning scale capture the more abstract meaning-making motivation related to one’s life, rather than more concrete need to make sense of the world and one’s actions.

Drawing on the conceptualization of the lower, more specific meaning type, Cantarero et al. (2019, 2021) proposed that people differ in the need to make sense of the world. To assess the corresponding individual differences, they developed the Need for Sense-Making Scale (NSM). The need for sense-making is understood as the desire to find reliable connections between actions, objects and events, which is essential to move about in the environment effectively. It is conceptualized as a personal resource as it elevates the chances of achieving a sense of meaning. Cantarero et al. (2021) found that need for sense-making related positively to openness to experience, self-esteem, and internal locus of control. Need for sense-making related to searching for meaning in a task, which was linked to perceiving the task as more meaningful and related to better task performance. Similarly, individual differences in need for sense-making were related to work engagement through changes in search for and the perceived presence of meaningful work. Additionally, in supplementary work (Cantarero et al., 2021, Study S2) these researchers showed that NSM is positively related to both searching and presence of meaning in life. The NSM measures the appraisal of and general tendency to engage in sense-making processes; this includes the valuation of search as well as the valuation of meaning as an experience. There is thus a growing body of evidence suggesting that it is worth taking into account individual differences in investigating motivation to make sense of what surrounds us.

## Need for sense-making and basic psychological needs

Although some have proposed that the need for sense-making is an important, core human motivation, it has thus far not been linked to basic psychological needs. According to self-determination theory, three basic psychological needs (BPN) are crucial to psychological functioning: autonomy, relatedness, and competence (Deci and Ryan, 2000; Vansteenkiste et al., 2020). The need for autonomy is related to engaging in activities that one chooses out of their volition. The need for relatedness holds that feeling connected to important others is of fundamental importance to individuals. Finally, the need for competence refers to the feeling of effectiveness in how one deals with their environment (Vansteenkiste et al., 2020). Satisfaction of the needs was found to relate positively to psychological well-being and negatively to experienced stress (e.g., Reis et al., 2000; Cantarero et al., 2021a). Additionally, recent research showed that satisfaction of the need for autonomy and the need for relatedness were linked to meaningful work indirectly through autonomous motivation, and need for competence was related to meaningful work directly (Autin et al., 2021).

Need for sense-making was not conceptualized as indicating whether the need is satisfied or frustrated. Those with high levels of the need value meaning highly, irrespective of the context. High levels of need for sense-making thus indicate that sense-making is an important motivation to an individual, rather than that the need is satiated. Similarly, for example, to the need to belong (Leary et al., 2013). However, given that NSM is understood as a personal resource that is beneficial to the functioning of individuals, it should be positively related to the satisfaction of BPN. More specifically, we expected that need for sense-making would relate positively to the satisfaction of the needs for autonomy, relatedness, and competence. Additionally, frustration of the needs relates to ill-being (e.g., Bartholomew et al., 2011). We expected that NSM is related negatively to the frustration of the three needs.

Previous studies tested and showed good psychometric properties of the need for sense-making scale (NSM). However, with 29 items, the scale is rather long, which may contribute to the unnecessarily extensive time that participants spend on filling in this one instrument. Besides pure practical reasons, a long scale can also unintendedly contribute to higher levels of fatigue in participants. Accordingly, the aim of this research was to test a shorter version of the NSM scale and to test how the need for sense-making relates to basic psychological needs, going beyond the earlier research.

## Study overview

We conducted four studies to develop and evaluate the short version of the Need for Sense-Making Scale (Cantarero et al., 2021). In Study 1, we selected items most representative of the construct. A set of seven items met this criterion (Appendix 1). In Study 2 through 4, we administered the Need for Sense-Making Scale Short Form (NSM-SF), alongside other measures of relevant constructs (e.g., basic psychological needs, search and presence of meaning in life), to test its factorial structure, as well as convergent and divergent validity. The studies were approved by the Faculty Research Ethics Committee and were conducted in accordance with APA guidelines and the Helsinki Declaration of Human Right. All the data files are available at https://osf.io/8s95h/?view_only=55e20ba099384ad28d2dcd35f32a4c2a.

## Study 1

The aim of Study 1 was to identify items for the NSM-SF. To this end, we considered all the data from the studies presenting the long version of the scale in English reported in Cantarero et al. (2021a). Additionally, we included the data from one new study (Cantarero, 2022), which allowed us to draw reliable conclusions due to the relatively large sample.

### Participants

The analyzed sample consisted of five hundred eighty-two participants (287 women, 293 men, two undisclosed). Age ranged from 18 to 70 (*M*_age_ = 33.86, *SD*_age_ = 12.52). One hundred and forty-seven participants were from the United Kingdom and 435 participants were from the United States. United Kingdom participants were university students who took part in the research without remuneration. US participants were MTurk workers who received financial remuneration for participation.

### Results and discussion

Similar to Cantarero et al. (2021) we performed diagonally weighted least squares confirmatory factor analysis of the full scale, which supported the unifactorial structure of the scale; the model had a moderate fit to the data χ^2^/df = 5.21; RMSEA = 0.09, 90% *CI* = [0.083, 0.091], SRMR = 0.10, GFI = 0.93, though χ^2^/df exceeded the recommended 5.00.^2^

We next reviewed the content of the items to select a subset for the short version of the scale. We selected these such as to retain translational validity (i.e., construct and face validity) in light of our theoretical definition of need for sense-making. We sought to establish reliability, criterion validity, and appropriate factor structure in separate steps. We noticed that some items overlapped strongly in content (e.g., “I don’t like it when things serve no purpose” and “Doing pointless activities doesn’t bother me”). To avoid biased construct representation that might occur by having some items being much more similar in content to each other than others, we excluded redundant items to select only those that differed substantially. We chose one item that described reactions to novelty and discrepancy (*When I am in a new situation, I try to find meaning in it*), persistence in searching for meaning (*I tend to search for meaning of discrepant situations until I find it*), general preference of meaningful vs. meaningless activities (*I prefer to do things that are meaningful*), positive affect related to experiencing meaningfulness (*When I make sense of a situation it is pleasant to me*), negative affect related to purposeless activities (*I don’t like it when things serve no purpose*), tendency to look for activities that are purposeful (*I search for activities that serve a purpose*) and we also decided to include one reverse coded item (*I don’t usually try to find purpose of things*).

A diagonally weighted least squares CFA on the resultant seven items evidenced adequate fit to the data, χ^2^/df = 1.03; RMSEA = 0.01, 90% *CI* = [0.000, 0.042], SRMR = 0.04, GFI = 0.99. All factor loadings exceed 0.35 with *p* < 0.001 (Table 1).

**TABLE 1:** Standardized factor loadings based on confirmatory factor analysis for seven items of the Need for Sense-Making Scale (*N* = 583).

Item	Factor loading
(1) I search for activities that serve a purpose	0.71
(2) When I make sense of a situation it is pleasant to me	0.71
(3) I prefer to do things that are meaningful	0.66
(4) I don’t like it when things serve no purpose	0.57
(5) I don’t usually try to find purpose of things	0.35
(6) When I am in a new situation I try to find meaning in it	0.80
(7) I tend to search for meaning of discrepant situations until I find it	0.72

Both long and short versions of the scale had high internal consistency, α = 0.91 and α = 0.82, respectively. The two versions of the scale were highly correlated *r*(582) = 0.91, *p* < 0.001.

The results of this study gave initial support for the 7-item solution of the NSM. We found that the long and short versions share similar psychometric properties with respect to internal consistency and are highly correlated, which suggests that the short version can be equivalent to the longer one.

## Study 2

The aim of Study 2 was to further test the unifactorial structure of the NSM-SF. We also refined the wording of one of the items. Specifically, the double negation in item 23 (*I don’t like it when things serve no purpose*) was changed to *I like it when things serve a purpose*. We conducted a confirmatory factor analysis to test the underlying dimensional structure of the scale relying on a new sample of participants.

### Method

#### Participants and recruitment

Participants were 287 participants MTurk workers residing in the United States, who received $1.10 for taking part in the study.^3^ The sample consisted of 143 women, 136 men, and one other; seven individuals did not disclose their gender. Ages ranged from 20 through 74 (*M*_age_ = 40.20, *SD*_age_ = 11.01).

#### Procedure and materials

After giving their informed consent, participants completed the revised NSM-SF using a seven-point Likert scale (1 = *not at all*, 7 = *very much*). At the end of the study, we gathered demographic data and debriefed participants.

### Results and discussion

We tested whether the data corresponded to the unifactorial model. A diagonally weighted least squares confirmatory factor analysis (CFA) yielded good fit, χ^2^/df = 0.64; RMSEA = 0.001, 90% *CI* = [0.000, 0.035], SRMR = 0.05, GFI = 0.99. All of the standardized regression weights were above 0.50 with *p* < 0.001 (Table 2).

**TABLE 2:** Standardized factor loadings based on confirmatory factor analysis for seven items of the Need for Sense-Making Scale (*N* = 287, Study 2).

Item	Factor loading
(1) I search for activities that serve a purpose	0.80
(2) When I make sense of a situation it is pleasant to me	0.69
(3) I prefer to do things that are meaningful	0.83
(4) I like it when things serve a purpose	0.82
(5) I don’t usually try to find purpose of things	0.50
(6) When I am in a new situation I try to find meaning in it	0.79
(7) I tend to search for meaning of discrepant situations until I find it	0.63

Additionally, we analyzed the internal consistency of the scale in this sample. The results indicated high internal consistency of the NSM-SF (α = 0.88). These results confirm the unidimensional structure of the scale and its internal reliability.

## Study 3

The aim of Study 3 was to analyze the test-retest reliability of the short Need for Sense-Making Scale. The original scale measures relatively stable individual differences. Here we tested if, as for the original scale, scores measured by the short version of the scale were stable over time.

### Method

#### Participants and procedure

We gathered data from 65 MTurk workers residing in the United States (35 women) *M*_age_ = 42.65, *SD* = 11.52 (age ranged from 22 to 65), who completed the NSM-SF twice with a break of above 6 weeks between the two measurements (42 days). Participation was rewarded with $2.10. Participants completed the NSM-SF and then provided demographic data. The overall sample size yields corresponding power in excess of 1 – β = 0.95, for lower and upper critical *r* of ∣0.24∣ (α = 0.05, two-tailed).

### Results and discussion

Internal consistency of the scales measured with Cronbach’s α for both measurements was high (α_*T*1_ = 0.88, α_*T*2_ = 0.87). The test-retest reliability of the scale was good: Time 1 and time 2 scores correlated *r*(65) = 0.74, *p* < 0.001. These results show good test-retest reliability of the scale, especially considering the relatively long period between the test and the retest. This further indicates that the short version of the scale demonstrates similar properties to the original version.

## Study 4

In Study 4, we examined the convergent and divergent validity of the scale. We anticipated moderate and positive associations between need for sense-making and the satisfaction of the basic psychological needs. We also expected that need for sense-making related moderately negatively to the frustration of basic psychological needs. Similarly, as in previous studies, we expected that need for sense-making related positively to both presence and search for meaning in life (Cantarero et al., 2021). Should these relationships emerge then this would speak to the convergent validity of the short NSM. To test the discriminant validity of the scale, we examined if the construct related to the tendency to anthropomorphize. We had no reason to believe that the two constructs, need for sense-making and anthropomorphizing, are indeed related.

### Method

#### Participants and recruitment

We aimed at maximizing the number of participants we could reach within the possibility we had to conduct the study. There were 308 MTurk workers residing in the United States (111 women, 190 men, one other, six unstated) who took part in the online study. Age ranged from 18 through 72 (*M*_age_ = 38.82, *SD*_age_ = 11.65). Participation in the study was compensated with $1. We performed a sensitivity power analysis for the lowest correlation between the variables of interest in the study. The overall sample size yields corresponding power in excess of 1 – β = 0.95 for *r* = 0.19 (α = 0.05, two-tailed) with lower and upper critical *r* = ∣0.11∣.

#### Procedure and materials

Participants completed four scales that were presented in random order. They completed the 10-item Meaning in Life Questionnaire (MLQ, Steger et al., 2006), with answers ranging from 1 = *absolutely untrue*, 7 = *absolutely true*. The scale consists of two subscales: searching for meaning in life (α = 0.88, e.g., *I am seeking a purpose or mission for my life*) and presence of meaning in life (α = 0.93, e.g., *I have discovered a satisfying life purpose*). We also asked participants to fill in the 24-item Basic Psychological Need Satisfaction and Frustration Scale (BPNSF, Chen et al., 2015) with answers ranging from 1 = *completely untrue*, 5 = *completely true*. The scale consists of six subscales: autonomy satisfaction (α = 0.82, e.g., *I feel a sense of choice and freedom in the things I undertake*), autonomy frustration (α = 0.86, e.g., *My daily activities feel like a chain of obligations*), relatedness satisfaction (α = 0.85, e.g., *I feel close and connected with other people who are important to me*), relatedness frustration (α = 0.90, e.g., *I feel the relationships I have are just superficial*), competence satisfaction (α = 0.87, e.g., *I feel I can successfully complete difficult tasks*) and competence frustration (α = 0.92, e.g., *I feel like a failure because of the mistakes I make*). Participants filled in the 30-item Individual Differences in Anthropomorphism Questionnaire (IDAQ, Waytz et al., 2010), as well, with ratings between 0 = *not at all* to 10 = *very much*. The scale includes a 15-item measure of anthropomorphization (α = 0.94, e.g., *To what extent does a cheetah experience emotions?*) and 15 non-diagnostic items. Finally, participants also filled in the NSM-SF (α = 0.83).

### Results and discussion

We conducted a correlation analysis (Table 3). The results showed that the need for sense-making measured with the seven items was positively and moderately related to the satisfaction of basic psychological needs. It was related negatively to the frustration of the needs. Additionally, similarly to previous findings with the long version of the NSM (Cantarero et al., 2021), it was positively related to searching and presence of meaning in life. There was no significant relationship between need for sense-making and the tendency to anthropomorphize. These results confirm the convergent and discriminant validity of the short version of the NSM scale.

**TABLE 3:** Correlation matrix for Study 4.

Variable	1	2	3	4	5	6	7	8	9
(1) Need for sense-making	–								
(2) Autonomy satisfaction	0.42***	–							
(3) Autonomy frustration	−0.15**	−0.39***	–						
(4) Relatedness satisfaction	0.43***	0.50***	−0.43***	–					
(5) Relatedness frustration	−0.24***	−0.24***	0.70***	−0.52***	–				
(6) Competence satisfaction	0.43***	0.60***	−0.37***	0.53***	−0.28**	–			
(7) Competence frustration	−0.24***	−0.38***	0.74***	−0.46***	0.74***	−0.58***	–		
(8) Presence of meaning in life	0.39**	0.53***	−0.31***	0.48***	−0.20***	0.59***	−0.46***	–	
(9) Searching for meaning in life	0.22***	−0.16**	0.45***	−0.20**	0.45***	−0.19**	0.48***	−0.33***	–
(10) Anthropomorphization	–0.07	–0.02	0.46***	−0.13*	0.61***	–0.07	0.52***	0.04	0.36***

*p < 0.05, **p < 0.01, ***p < 0.001.

## General discussion

One of our aims was to develop a short form of the need for sense-making scale. To this end, we tested the psychometric properties of the Need for Sense-Making scale Short Form by means of four studies. In Study 1, we chose seven items that formed NSM-SF and showed that the short version shows similar internal consistency as the longer version and that the two versions of the scale are highly correlated. In Study 2, we confirmed the unifactorial structure of the scale and its’ high internal consistency. Results of Study 3 indicated good test-retest reliability of the NSM-SF. Taken together, we found that the short version of the scale is reliable. It presents the same pattern of results with related constructs as the original version of the scale as it relates positively yet weakly to both searching and presence of meaning in life.

We also aimed to test the relationship between need for sense-making and basic psychological needs. Study 4 showed that need for sense-making relates positively to the satisfaction of basic psychological needs, and it relates negatively to the frustration of the needs. The finding that individual differences in need for sense-making correlate positively with the three facets of self-determination (autonomy, relatedness, and competence) and negatively with their frustration is important not just for psychometric reasons but also from a theoretical vantage point. Complementing the established finding that *having* a sense of meaning contributes to human well-being (Heintzelman, 2018), our results showcase that those who benefit from having satisfied their basic psychological needs also possess a prominent *need* to make sense of the world. While we should, of course, be cautious about the causal relation of these variables, it casts meaning as a phenomenon that may be beneficial to possess *and* to require.

Research suggests that people who are in search of a sense of meaning in their lives tend to be worse off in terms of, for example, their social relatedness and self-acceptance (Steger et al., 2008). Interestingly, while search for meaning in life and the need for sense-making are (modestly) positively correlated, they thus exhibit partly opposite associations with well-being outcomes. Why might this be the case? A possibility is that some who find themselves in perpetual search for meaning are, in fact, desperately lacking it; indeed, searching for meaning in its absence comes with reduced life satisfaction (Steger et al., 2011), and those high in search for meaning tend to be somewhat lower in perceived presence of meaning (Steger et al., 2008; Van Tilburg and Igou, 2016; Van Tilburg et al., 2019). Those who set high sense-making aspirations, however, may instead be more often successful in its attainment than those who are not, as evident from the correlations between need for sense-making with *both* the perceived presence of meaning in life and the search for it.

The research presented here is one of the first steps in considering the need for sense-making as part of meaning-making models and theories of psychological needs. Although some researchers (e.g., Baumeister, 1991; Frankl, 2006) have argued that people have a need to make sense of the world and that this need is one of the essential human motivations, surprisingly, it has not been included in models that focus on core psychological needs. We hope that, given the growing interest in meaning-making motivation, our tool will enable researchers to examine the need for sense-making as a candidate in the pantheon of human motivation.

## Limitations and directions of future research

One of the limitations of the presented research is that it was conducted mainly with MTurk participants. It would be worthwhile to test the scale in other populations. Furthermore, in Study 2, we refined the wording of one item to make it fit better with the other items in the scale, and the studies that followed used this refined version. Although the studies we present show that NSM and NSM-SF overlap, because of the change in wording in one item, it might be more accurate to treat NSM-SF as a separate scale rather than a shorter version of NSM. We also acknowledge that the studies we present do not cover all possible tests of the validity of the scale. Additional research could focus on the predictive power of NSM-SF or compare it with similar constructs.

Future studies should also examine the role of the need for sense-making and meaningful work in more detail. Cantarero et al. (2021) suggest that NSM is a personal resource that elevates the chances of finding a sense of meaning. Applying this to the work context, they found that NSM was positively related to the search for and presence of meaning at work, which in turn was associated with work engagement. The latter was found to be beneficial for employees and organizations (Schaufeli and Bakker, 2014), as it is linked to better task performance, higher job satisfaction and financial benefits (Bakker and Albrecht, 2018). Therefore, NSM can play an important role in work-related meaning-making processes. For example, Cantarero et al. (2021b) found that meaning interventions enhanced the experience of meaningful work and, consequently, work engagement. It would be interesting to test if individual differences in need for sense-making moderate the effect. In principle, in activities where sense-making matters, the scale can test differences between people and, thus, the way they understand and engage in a situation. This quality might also predict more generally how people cope with threats, conflicts, and other forms of psychological challenges, which opens possible new research paths.

To sum up, this study offers a valid instrument to measure need for sense-making and is the first to show how need for sense-making relates to basic psychological needs.

## Data availability statement

The datasets presented in this study can be found in online repositories. The names of the repository/repositories and accession number(s) can be found below: https://osf.io/8s95h/?view_only=55e20ba099384ad28d2dcd35f32a4c2a.

## Ethics statement

The studies involving human participants were reviewed and approved by Faculty of Psychology in Wroclaw Committee of Ethics of Scientific Research at SWPS University, number 06/P/04/2020. The patients/participants provided their written informed consent to participate in this study.

## Author contributions

KC, WT, and AG designed the studies. KC gathered the data and conducted the analysis. All authors contributed to the article and approved the submitted version.

## Footnotes

1 We define “sense-making” and “meaning-making” as finding reliable connections between objects and use the two terms interchangeably throughout the manuscript. We define “meaningful” as having reliable connections between object, events or situations.

2 Standardized factor loadings of the long version of the scale are presented in Supplementary Table S1 in Supplementary Materials.

3 The scale was added at the beginning of a correlational study that focused on basic psychological needs and well-being.

## Appendix 1

Please indicate how much each of the following statements reflects how you typically are, using the scale provided.

Not at all 1 2 3 4 5 6 7 Very much

**Table:**

(1) I search for activities that serve a purpose	1	2	3	4	5	6	7
(2) When I make sense of a situation it is pleasant to me	1	2	3	4	5	6	7
(3) I prefer to do things that are meaningful	1	2	3	4	5	6	7
(4) I like it when things serve a purpose	1	2	3	4	5	6	7
(5) I don’t usually try to find purpose of things	1	2	3	4	5	6	7
(6) When I am in a new situation I try to find meaning in it	1	2	3	4	5	6	7
(7) I tend to search for meaning of discrepant situations until I find it	1	2	3	4	5	6	7

Reverse coded item: 5.

## References

[B1] Abeyta A. A. Routledge C. (2018). The need for meaning and religiosity: An individual differences approach to assessing existential needs and the relation with religious commitment, beliefs, and experiences. *Personal. Individ. Diff.* 123 6–13. 10.1016/j.paid.2017.10.038

[B2] Autin K. L. Herdt M. E. Garcia R. G. Ezema G. N. (2021). Basic Psychological Need Satisfaction, Autonomous Motivation, and Meaningful Work: A Self-Determination Theory Perspective. *J. Career Assess.* 30 78–93. 10.1177/10690727211018647

[B3] Bakker A. B. Albrecht S. (2018). Work engagement: Current trends. *Career Dev. Int.* 23 4–11.

[B4] Bartholomew K. J. Ntoumanis N. Ryan R. M. Bosch J. A. Thøgersen-Ntoumani C. (2011). Self-determination theory and diminished functioning: The role of interpersonal control and psychological need thwarting. *Personal. Soc. Psychol. Bull.* 37 1459–1473. 10.1177/0146167211413125 21700794

[B5] Baumeister R. F. (1991). *Meanings in Life.* New York: Guilford.

[B6] Byrka K. Cantarero K. Dolinski D. Van Tilburg W. (2021). Consequences of Sisyphean Efforts: Meaningless Effort decreases motivation to engage in subsequent conservation behaviors through disappointment. *Sustainability* 13:5716. 10.3390/su13105716

[B7] Cantarero K. (2022). Need for sense-Making and Turnover Intentions. On Why Some people Need Their Work to be meaningful to not search for a new one. Unpublished manuscript.

[B8] Cantarero K. Van Tilburg W. A. Gąsiorowska A. Wojciszke B. (2021). The need for sense-making as a personal resource: Conceptualization and scale development. *Curr. Psychol.* 1–12. 10.1007/s12144-021-01637-3

[B9] Cantarero K. Van Tilburg W. A. Smoktunowicz E. (2021a). Affirming basic psychological needs promotes mental well-being during the COVID-19 outbreak. *Soc. Psychol. Personal. Sci.* 12 821–828. 10.1177/1948550620942708

[B10] Cantarero K. Van Tilburg W. Smoktunowicz E. (2021b). Other-(vs. self-) oriented meaning interventions enhance momentary work engagement through changes in work meaningfulness. *J. Counsel. Psychol.* 69 443–451. 10.1037/cou0000594 34780204

[B11] Cantarero K. Van Tilburg W. A. P. Kuzma B. Gasiorowska A. Wojciszke B. (2019). Some people probably need to make more sense: An exploratory study on individual differences and the need for sense-making. *Polish Psychol. Bull.* 50 114–118. 10.24425/ppb.2019.126026

[B12] Chen B. Vansteenkiste M. Beyers W. Boone L. Deci E. L. Van der Kaap-Deeder J. (2015). Basic psychological need satisfaction, need frustration, and need strength across four cultures. *Motiv.Emot.* 39, 216–236. 10.1007/s11031-014-9450-1

[B13] Deci E. L. Ryan R. M. (2000). The “what” and “why” of goal pursuits: Human needs and the self-determination of behavior. *Psychol. Inquiry* 11 227–268.

[B14] Frankl V. (2006). *Men’s Search for Meaning.* Boston: Beacon Press.

[B15] Graeupner D. Coman A. (2017). The dark side of meaning-making: How social exclusion leads to superstitious thinking. *J. Exp. Soc. Psychol.* 69 218–222. 10.1016/j.jesp.2016.10.003

[B16] Heintzelman S. J. (2018). *Eudaimonia in the Contemporary Science of Subjective Well-Being: Psychological Well-Being, Self-Determination, and Meaning in Life.* Salt Lake City, UT: DEF Publishers.

[B17] Iwasaki Y. Messina E. S. Hopper T. (2018). The role of leisure in meaning-making and engagement with life. *J. Positive Psychol.* 13 29–35. 10.1080/17439760.2017.1374443

[B18] Leary M. R. Kelly K. M. Cottrell C. A. Schreindorfer L. S. (2013). Construct validity of the need to belong scale: Mapping the nomological network. *J. Personal. Assess.* 95 610–624. 10.1080/00223891.2013.819511 23905716

[B19] May D. R. Gilson R. L. Harter L. M. (2004). The psychological conditions of meaningfulness, safety and availability and the engagement of the human spirit at work. *J. Occup. Organ. Psychol.* 77 11–37. 10.1348/096317904322915892

[B20] Petrou P. Bakker A. B. van den Heuvel M. (2017). Weekly job crafting and leisure crafting: Implications for meaning-making and work engagement. *J. Occup. Organ. Psychol.* 90 129–152. 10.1111/joop.12160

[B21] Reis H. T. Sheldon K. M. Gable S. L. Roscoe J. Ryan R. M. (2000). Daily well-being: The role of autonomy, competence, and relatedness. *Personal. Soc. Psychol. Bull.* 26 419–435. 10.1177/0146167200266002

[B22] Schaufeli W. B. Bakker A. B. (2014). “Defining and measuring work engagement: Bringing clarity to the concept,” in *Work engagement: A Handbook of Essential Theory and Research*, eds Bakker A. B. Leiter M. P. (New York: Psychology Press), 10–24.

[B23] Steger M. F. Frazier P. Oishi S. Kaler M. (2006). The meaning in life questionnaire: Assessing the presence of and search for meaning in life. *J. Counsel. Psychol.* 53 80–93. 10.1037/0022-0167.53.1.80

[B24] Steger M. F. Kashdan T. B. Sullivan B. A. Lorentz D. (2008). Understanding the search for meaning in life: Personality, cognitive style, and the dynamic between seeking and experiencing meaning. *J. Personal.* 76 199–228. 10.1111/j.1467-6494.2007.00484.x 18331281

[B25] Steger M. F. Oishi S. Kesebir P. (2011). Is a life without meaning satisfying? The moderating role of the search for meaning in satisfaction with life judgments. *Positive Psychol.* 6 173–180. 10.1080/17439760.2011.569171

[B26] Updegraff J. A. Silver R. C. Holman E. A. (2008). Searching for and finding meaning in collective trauma: Results from a national longitudinal study of the 9/11 terrorist attacks. *J. Personal. Soc. Psychol.* 95:709. 10.1037/0022-3514.95.3.709 18729704 PMC2617710

[B27] Van Tilburg W. A. Igou E. R. (2016). Going to political extremes in response to boredom. *Eur. J. Soc. Psychol.* 46 687–699. 10.1002/ejsp.2205

[B28] Van Tilburg W. A. P. Igou E. R. Maher P. J. Moynihan A. B. Martin D. G. (2019). Bored like Hell: Religiosity reduces boredom and tempers the quest for meaning. *Emotion* 19 255–269. 10.1037/emo0000439 29697990

[B29] Vansteenkiste M. Ryan R. M. Soenens B. (2020). Basic psychological need theory: Advancements, critical themes, and future directions. *Motiv. Emot.* 44 1–31. 10.1007/s11031-019-09818-1

[B30] Walsh F. (2020). Loss and resilience in the time of COVID-19: Meaning making, hope, and transcendence. *Fam. Proc.* 59 898–911. 10.1111/famp.12588 32678915 PMC7405214

[B31] Waytz A. Cacioppo J. Epley N. (2010). Who sees human? The stability and importance of individual differences in anthropomorphism. *Perspect. Psychol. Sci.* 5, 219–232. 10.1177/1745691610369336 24839457 PMC4021380

