# Supplementary material for: The efficient measurement of individual differences in meaning motivation: The need for sense-making short form
Source: Front Psychol. 2022 Aug 18;13:945692. doi: 10.3389/fpsyg.2022.945692 (PMC9435525; doi:10.3389/fpsyg.2022.945692)
Supplement: Supplementary file 1 [file Table_1.DOCX]

**Supplementary Materials**

**The Efficient Measurement of Individual Differences in Meaning Motivation: The Need for Sense-Making Short Form (NSM-SF)**

Table S1

*Standardized regression weighs for the 29 items Need for Sense-Making Scale (N = 582). All factor loadings were at p < .001.*

|  | Factor loadings |
| --- | --- |
| I like it when I stop feeling bored | .27 |
| I often get engaged in meaningful activities | .43 |
| When I’m in a new situation I can’t avoid searching meaning in it | .66 |
| Usually when I can’t make sense of a situation I feel upset | .40 |
| When I do something that is meaningless I feel bad | .44 |
| When I do something that is meaningful I feel good | .45 |
| When I can’t find the purpose of a situation it’s unpleasant | .54 |
| Even if a new situation is of little importance I would still try to find purpose in it | .70 |
| I don’t like to feel bored | .34 |
| **I prefer to do things that are meaningful** | .61 |
| **When I am in a new situation I try to find meaning in it** | .76 |
| **When I make sense of a situation it is pleasant to me** | .67 |
| Usually, when I do something that is meaningless I want to switch to do something else | .59 |
| Usually, when I find a discrepant situation I try to make sense of it | .68 |
| When I feel bored I quickly try to do something to change it | .43 |
| When things have no meaning it doesn’t bother me at all | .27 |
| I don’t pursue purposeful activities | .22 |
| **I search for activities that serve a purpose** | .66 |
| I often engage myself in making sense of different situations | .72 |
| **I tend to search for meaning of discrepant situations until I find it** | .76 |
| I don’t mind feeling bored | .23 |
| I avoid situations that make no sense | .55 |
| **I don’t like it when things serve no purpose** | .64 |
| **I don’t usually try to find purpose of things** | .37 |
| Doing pointless activities doesn’t bother me | .37 |
| When I’m in an unexpected situation, the first thing that I want to do is to find meaning in it | .67 |
| When I evaluate an activity as pointless I lose interest in doing it | .48 |
| I often wonder what are the relationships between things | .56 |
| I avoid doing boring things | .46 |

*Note:* Items selected for the NSM-SF are in bold.
